# Correction: Li, P., et al. Research on Erosion-Corrosion Rate of 304 Stainless Steel in Acidic Slurry via Experimental Design Method. *Materials* 2019, *12*, 2330

**DOI:** 10.3390/ma12193181

**Published:** 2019-09-28

**Authors:** Ping Li, Yanjie Zhao, Libo Wang

**Affiliations:** 1 Henan Key Laboratory of Materials on Deep-Earth Engineering, School of Materials Science and Engineering, Henan Polytechnic University, Jiaozuo 454000, China; yanjiezhao90@163.com (Y.Z.); wanglibo537@hpu.edu.cn (L.W.); 2 State Key Laboratory of Solid Lubrication, Lanzhou Institute of Chemical Physics, Chinese Academy of Sciences, Lanzhou 730000, China; 3 Henan International Joint Research Laboratory for High-Performance Light Metallic Materials and Numerical Simulations, Jiaozuo 454000, China

The authors wish to make the following corrections to this paper [1]:

We found an error in 2.3. Experimental Methodology, i.e., on page 4 of 11, lines 19–20, where the sentence “The average size and mass fraction of the sands were 20~40 mesh and 10%” should be “The average size and mass fraction of the sands were 20~40 mesh and 5%”.

The authors would like to apologize for any inconvenience caused to the readers by this change.
